# Molecular Characterization and Expression Analysis of Intercellular Adhesion Molecule-1 (ICAM-1) Genes in Rainbow Trout (*Oncorhynchus mykiss*) in Response to Viral, Bacterial and Parasitic Challenge

**DOI:** 10.3389/fimmu.2021.704224

**Published:** 2021-08-20

**Authors:** Xue Zhai, Wei-Guang Kong, Gao-Feng Cheng, Jia-Feng Cao, Fen Dong, Guang-Kun Han, Yan-Ling Song, Chuan-Jie Qin, Zhen Xu

**Affiliations:** ^1^Department of Aquatic Animal Medicine, College of Fisheries, Huazhong Agricultural University, Wuhan, China; ^2^State Key Laboratory of Freshwater Ecology and Biotechnology, Center for Fish Biology and Fishery Biotechnology, Institute of Hydrobiology, Chinese Academy of Sciences, Wuhan, China; ^3^Department of Life Science, Key Laboratory of Sichuan Province for Conservation and Utilization of Fishes Resources in the Upper Reaches of the Yangtze River, Neijiang Normal University, Neijiang, China

**Keywords:** intercellular adhesion molecule-1, rainbow trout, infectious hematopoietic necrosis virus, *Flavobacterium columnare G_4_*, *Ichthyophthirius multifiliis*, mucosal immune response

## Abstract

The intercellular adhesion molecule-1 (ICAM-1), known as CD54, is a transmembrane cell surface glycoprotein that interacts with two integrins (i.e., LFA-1 and Mac-l) important for trans-endothelial migration of leukocytes. The level of ICAM-1 expression is upregulated in response to some inflammatory stimulations, including pathogen infection and proinflammatory cytokines. Yet, to date, our knowledge regarding the functional role of ICAM-1 in teleost fish remains largely unknown. In this study, we cloned and characterized the sequence of ICAM-1 in rainbow trout (*Oncorhynchus mykiss*) for the first time, which exhibited that the molecular features of ICAM-1 in fishes were relatively conserved compared with human ICAM-1. The transcriptional level of ICAM-1 was detected in 12 different tissues, and we found high expression of this gene in the head kidney, spleen, gills, skin, nose, and pharynx. Moreover, upon stimulation with infectious hematopoietic necrosis virus (IHNV), *Flavobacterium columnare* G_4_ (*F. columnare*), and *Ichthyophthirius multifiliis* (Ich) in rainbow trout, the morphological changes were observed in the skin and gills, and enhanced expression of ICAM-1 mRNA was detected both in the systemic and mucosal tissues. These results indicate that ICAM-1 may be implicated in the mucosal immune responses to viral, bacterial, and parasitic infections in teleost fish, meaning that ICAM-1 emerges as a master regulator of mucosal immune responses against pathogen infections in teleost fish.

## Introduction

Intercellular adhesion molecule-1 (ICAM-1), an inducible transmembrane glycoprotein, belongs to the immunoglobulin superfamily. Unlike ICAM-2 (1), which has only two immunoglobulin-like (Ig-like) domains in mammals, ICAM-1 consists of five distinct Ig-like domains, a transmembrane domain, and a short cytoplasmic tail (2, 3), and therefore its structure is more similar to ICAM-3 (CD50) (4). Additionally, the existence of cysteine residues located close to the cell membrane contributes to the formation of intermolecular disulfide bonds (3), implying that ICAM-1 exists as a dimer (2). Previous studies have demonstrated that ICAM-1 mainly binds to lymphocyte function-associated antigen 1 (LFA-1, CDlla/CD18) (5) and macrophage antigen 1 (Mac-l, CDllb/CD18) (6, 7) (i.e., two integrins belonging to the β2 subfamily) as receptors that are essential for cell–cell and cell–matrix adhesive interactions and activation of signal-transduction into the cell pathways (8). Although LFA-1 is the common receptor of ICAM-1, ICAM-2, and ICAM-3, the affinity of LFA-1 to bind the ICAM-1 is higher than that of ICAM-2 and ICAM-3 (9). Integrins are expressed by leukocyte and specifically bind to different Ig-like domains of ICAM-1. For instance, LFA-1 primarily binds to the first Ig-like domain, and Mac-1 binds to the third Ig-like domain of ICAM-1 (8), suggesting that ICAM-1 has the potential to bind both LFA-1 and Mac-1 simultaneously. CD43, also known as *sialophorin*, a lesser-known membrane receptor for ICAM-1, facilitates the adhesion of Th17 cells to ICAM-1 and modulates apical and trans-endothelial migration (10). In addition, the interaction between ICAM-1 and CD43 plays an important role in the adhesion and invasion of tumor cells to peritoneum (11). Of note, ICAM-1 also serves as pathogen receptors, such as a sequestration receptor for malarial parasite *Plasmodium falciparum* (12), human rhinovirus (13), and Coxsackievirus A21 (14, 15) and as a coreceptor for human immunodeficiency virus-1 (HIV-1) (16).

ICAM-1 is expressed at low basal levels in numerous cell types including immune cells, endothelial cells, and epithelial cells, but it is upregulated under inflammatory conditions (8, 17). The level of ICAM-1 expression can be highly induced by various proinflammatory cytokines, such as tumor necrosis factor-α (TNF-α), interleukin-1β (IL-1β), and interferon-γ (IFN-γ), and inhibited by glucocorticoids (18). Interestingly, the degree of ICAM-1 expression induced by cytokines differs depending on cell types. For instance, a robust upregulation of ICAM-1 in intestinal endothelial cells was induced by TNF-α or IL-1β; however, in epithelial cells, IFN-γ could induce a high expression of ICAM-1, but not TNF-α and LPS (8). ICAM-1 actively participates in leukocytes trans-endothelial migration to sites of inflammation and as a costimulatory molecule for T-cell activation; thus, ICAM-1 plays an important role in both innate and adaptive immune responses (19). Under inflammatory conditions, increased expression of ICAM-1 resulted in enhanced polymorphonuclear neutrophil (PMN) binding to the intestinal apical epithelium, which increases epithelial cell proliferation and promotes intestinal mucosal wound healing (20). Moreover, recent studies have demonstrated that ICAM-1, as a costimulatory molecule, delivers a costimulatory signal into T cells and eventually leads to the activation of T cells (5, 19, 21). In addition to the cellular adhesive function, ICAM-1 is known to transmit outside-in signals by cross-linking ICAM-1 on the cell surface, which can affect the permeability of endothelial cells. Many studies show that ICAM-1 cross-linking on the B cell surface contributes to the activation of src-kinase family members p53/p56lyn (22). Overall, ICAM-1 is involved in many physiologic processes, which include regulating leukocyte trafficking, promoting pathogen and dead cell clearance, and activation of T cells and B cells (8, 19, 21, 23).

To date, ICAM-1 has been cloned in many mammals, such as human (2), chimpanzee (24), and canine (25). Moreover, the roles played by ICAM-1 in defense against pathogens including virus, bacteria, and parasite have been largely investigated in mammals. In mouse airway epithelial cells, ICAM-1 acts as a critical regulator in clearance of *H. influenzae* assistance with neutrophil-mediated bacterial killing (26). The crucial role of ICAM-1 in resisting *M. avium* infection and granuloma formation has been confirmed in the spleen and liver of mice (27). ICAM-1 induced by human bronchial epithelial cells plays a critical role in modulating the influenza virus survival (28). Also, the role of ICAM-1 has been clarified in dendritic cell–mediated HIV-1 transmission to CD4(+) T cells (29). ICAM-1-mediated dependent cytoadherence is essential for construction of the model of malarial parasite *P. falciparum* (30). In teleost, the molecular characteristics of ICAM-1 and trans-endothelial migration of leukocytes were only studied in grass carp (*Ctenopharyngodon idella*) (31). Thus, compelling evidence for the existence of ICAM-1 and its roles in response to different pathogens are important to be investigated. Rainbow trout (*Oncorhynchus mykiss*) is a salmonid belonging to Salmonidae *Oncorhynchus* and is one of the most widely cultivated cold-water fish in the world. However, several pathogens such as virus, bacteria, and parasite invade frequently, thus posing a serious risk to the profitability and the development of trout aquaculture.

In this study, we cloned and characterized the sequence of ICAM-1 in *Oncorhynchus mykiss* for the first time. Moreover, the differential expression of ICAM-1 mRNA was assessed in different tissues of trout including mucosal tissues, non-mucosal tissues, and systemic tissues. Additionally, we investigated ICAM-1-mediated immune responses in rainbow trout challenged with viral, bacterial, and parasitic infections. Morphological changes were observed in the skin and gills of organisms infected with infectious hematopoietic necrosis virus (IHNV; i.e., a virus), *Flavobacterium columnare* G_4_ (*F. columnare*; i.e., a bacterium), and *Ichthyophthirius multifiliis* (Ich; i.e., a parasite), which coincided with changes in ICAM-1 gene expression in both systemic and mucosal tissues. Therefore, our findings provide important insights into the predominant role of ICAM-1 genes in the mucosal immune responses to viral, bacterial, and parasitic infections in teleost fish.

## Materials and Methods

### Fish Maintenance

Healthy rainbow trout (average body weight: 10–15 g) used for the experiment were purchased from Shiyan Green Agricultural Science and Technology Company (Hubei Province, China) and were kept in aerated aquarium tanks (temperature 15°C) and fed with commercial diet twice a day (9:00 a.m. and 5:00 p.m.). Fish were allowed to adjust to the holding tanks for 2 weeks prior to treatment. To minimize the effects of feeding on the level of gene expression, fish of control and infected groups were both terminated feeding for 48 h prior to sampling.

### Full-Length cDNA Cloning of ICAM-1 and Sequence Analysis

#### Cloning the Full-Length cDNA of ICAM-1

To obtain a cDNA amplification template for ICAM-1, we first extracted the total RNA from rainbow trout spleen using TRIzol reagent (Invitrogen, USA), and then 1 μg of total RNA was subjected to reverse transcription using a 5× All-In-One MasterMix with AccuRT Genomic DNA Removal Kit (Abm, Canada) according to the standard protocol. Based on obtained sequence of ICAM-1 from *Oncorhynchus mykiss* transcriptome database (accession number XM_021557903.2), gene-specific primers were designed to amplify the internal region of ICAM-1. The amplified DNA fragments were firstly isolated using a Gel Extraction Kit (Sangon Biotech, China) and determined by sequencing (TSINGKE, China). Full-length cDNA sequences of ICAM-1 were amplified by 3′-RACE and 5′-RACE using the SMARTer RACE cDNA Amplification Kit (Clontech, USA) following the manufacturer’s instructions. All primers mentioned above are listed in **Supplementary Table 1**.

#### Sequence Analysis

Nucleotide and deduced amino acid sequences of ICAM-1 were conducted with the Basic Local Alignment Search Tool (BLAST) of the National Center for Biotechnology Information (http://www.ncbi.mlm.nih.gov.blast/BLAST.cgi). The predicted amino acid sequences of ICAM-1 were performed using the DNASTAR software. Protein analysis and positions of the signal peptide were identified with ExPASy online tools (http://us.expasy.org/tools) and SMART online website (http://smart.embl-heidelberg.de/), respectively. Different species of ICAM cDNAs were obtained from Genbank databases, then the DNAMAN software was used to perform multiple alignments. The phylogenetic trees were constructed from the amino acid sequences of the ICAM-1 by MEGA7.0 using the neighbor-joining method. Next, the exon and intron organization of ICAM-1 in different teleost fishes and human was analyzed by comparing the coding sequences or mRNA sequences of ICAM-1 to associated genomic sequences.

### Challenge Experiment

#### Virus Enrichment and Infection

The EPC cell line was generously gifted by Prof. Xueqin Liu (College of Fisheries, Huazhong Agricultural University, Wuhan, China) and was maintained at 28°C in 5% CO_2_ atmosphere and maintained in minimum Eagle’s medium (MEM, Gibco, USA) supplemented with 10% fetal bovine serum (FBS, Gibco, USA), 100 μg/ml streptomycin, and 100 U/ml penicillin. The IHNV kindly provided by Prof. Hong Liu (Shenzhen Entry-Exit Inspection and Quarantine Bureau) was propagated in EPC cells cultured in MEM medium with 2% FBS at 15°C. After the extensive cytopathic effect (CPE), the EPC cells with IHNV were collected and repeated freezing and thawing three times for virus suspension. As for the viral titer, viral supernatant was made 10-fold serial dilutions (10^−1^–10^−10^), and then each dilution was added to six replicated wells in a 96-well plate bespread with EPC cells. The 50% tissue culture infectious doses (TCID50/ml) were calculated through the Spearman and Kärber algorithm (32). For the infection experiment, trout were immersed with a dose of 6 ml IHNV (1 × 10^9^ PFU ml^−1^) in 10 L aeration water for 2 h at 15°C. Then trout were transferred to the aquarium containing new aquatic water. As a control, the same number of trout were same treated with the MEM. After that, trout were randomly sampled for the collection of head kidney, spleen, skin, and gills at 1, 4, 7, 14, 21, and 28 d after challenge, respectively. Samples from control fish were taken at the same time intervals with same methods.

#### Trout Challenged With *F. columnare*

*Flavobacterium columnare* G4 strain labeled with a green fluorescent protein (GFP) was kindly gifted from Prof. Pin Nie (Hydrobiology Chinese Academy of Sciences) and routinely cultured in Shieh broth as described previously (33). For challenge, fish (~3–5 g) were bathed with *F. columnare* G_4_ strain at a final concentration of 1 × 10^6^ CFU ml^–1^ for 4 h at 16°C and then migrated into the aquarium containing new aquatic water. For control group, trout were immersed with Shieh broth using the same methods. Tissues including head kidney, spleen, skin, and gills were sampled at 1, 4, 7, 14, 21, and 28 d after challenge from infection and control groups.

#### Ich Parasite Isolation and Infection

Isolation of Ich parasite was performed as previously reported by us (34, 35). Fish were exposed to a single dose of ~5,000 theronts per fish for 3 h, and then migrated into the aquarium containing new aquatic water to obtain infected fish. Mock-infected (uninfected) fish were exposed to the same tank water but without the parasite. At 1, 4, 7, 14, 21, and 28 d after infection, fish were euthanized with an overdose of tricaine methanesulfonate (MS-222, Syndel), and tissue samples consisting of head kidney, spleen, skin, and gills were collected from infected and mock-infected fish.

### RNA Isolation and Quantitative Real-Time PCR Analysis

To study the mRNA expression pattern of ICAM-1 in different tissues, healthy rainbow trout were anesthetized with MS-222, and then tissues including the head kidney, spleen, gills, muscle, gut, stomach, skin, liver, nose, buccal mucosa, pharynx, and eye were collected. Total RNA was extracted from the tissues and then subjected to reverse transcription with the SuperScript first-strand synthesis system for RT-qPCR (Yeasen, China). The expression levels of elongation factor 1α (EF-1α, housekeeping gene) and ICAM-1 were detected by RT-qPCR using the EvaGreen 2× qPCR Master mix (Yeasen, China) by a 7500 qPCR system (Applied Biosystems), 10 μl qPCR reaction mixture containing 5 μl EvaGreen 2× qPCR Master mix, 1 μl of the cDNA, 0.15 μl each of forward and reverse primer, and 3.7 μl H_2_O was performed for amplifications. The qPCR conditions were: 95°C for 5 min, followed by 40 cycles at 95°C for 10 s and at 58°C for 30 s. Primer sequences can be found in **Supplementary Table 1**.

### Histology, Light Microscopy, and Immunofluorescence Microscopy Studies

To assess the morphological changes of skin and gills after infection of virus, bacteria, and parasite, histopathological examination was used in this experiment. Briefly, after dissected, the gills and skin of rainbow trout were fixed in 4% neutral buffered formalin overnight at 4°C, and then transferred to graded ethanol for dehydration and dimethylbenzene for vitrification. After that, the tissues were embedded in paraffin and cut in 5 μm thick sections with a rotary microtome (MICROM International GmbH, Germany). After being stained with conventional hematoxylin and eosin (HE), the sections were examined under the microscope (Olympus, BX53, Japan) using the Axiovision software to acquire and analyze images. For the immunofluorescence of *Flavobacterium columnare* labeled with a green fluorescent protein (GFP), the gill tissues were sampled and then embedded in optimal cutting compound (OCT, SAKURA, USA) for frozen sections with the freezing microtome (Leica). Then the sections were fixed in 4% neutral buffered formalin and stained with DAPI (4’, 6-diamidino-2-223 phenylindole; 1 μg/ml: Invitrogen) before mounting. All images were acquired and analyzed using an Olympus BX53 fluorescence microscope (Olympus) and the iVision-Mac scientific imaging processing software (Olympus).

### Statistical Analysis

Data were expressed given as the mean ± SEM and checked for normality and homogeneity of variances before statistical analysis. The statistical analysis was performed using an unpaired Student’s t-test (Prism version 6.0; GraphPad). *P < 0.05* was considered statistically significant.

## Results

### Identification and Analysis of ICAM-1 Gene From *Oncorhynchus mykiss*

**Figure 1:**
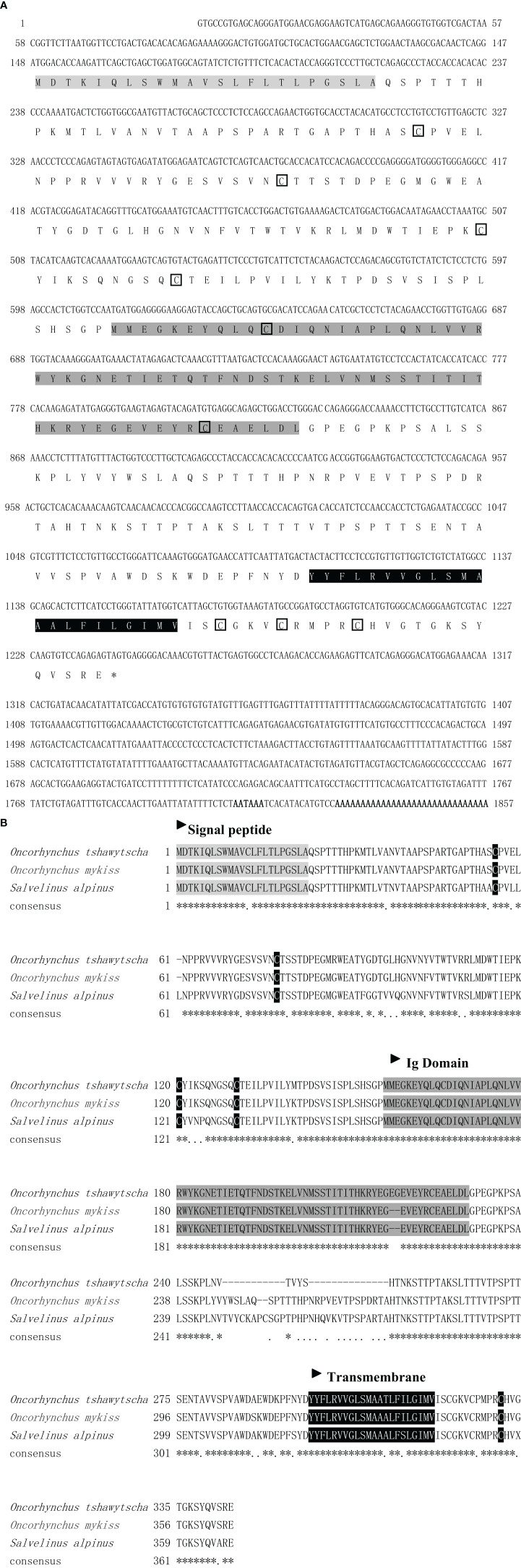
The nucleotide and deduced amino acid sequences **(A)** and multiple sequence alignment of deduced amino acid sequences **(B)** of ICAM-1 chain in rainbow trout and other teleost fish. Signal peptide is illumed with light gray. Cysteine is circled with the pane. The Ig domain is shaded in dark gray. The transmembrane is marked in black. *Indicates the similarity of the sequence.

The full-length sequence of ICAM-1 was of 1,958 bp and contained an open reading frame (ORF) of 1,095 bp, which encoded a predicted protein of 365 amino acids (**Figure 1A**). Analysis of sequence structure and multiple sequence alignment reveals that ICAM-1 contained signal peptide, Ig-like domain, transmembrane domain, and free cysteine residues similar with *Oncorhynchus tshawytscha* and *Salvelinus alpinus* (**Figure 1B**).

### Phylogenetic Analysis of ICAM-1 Gene

**Figure 2:**
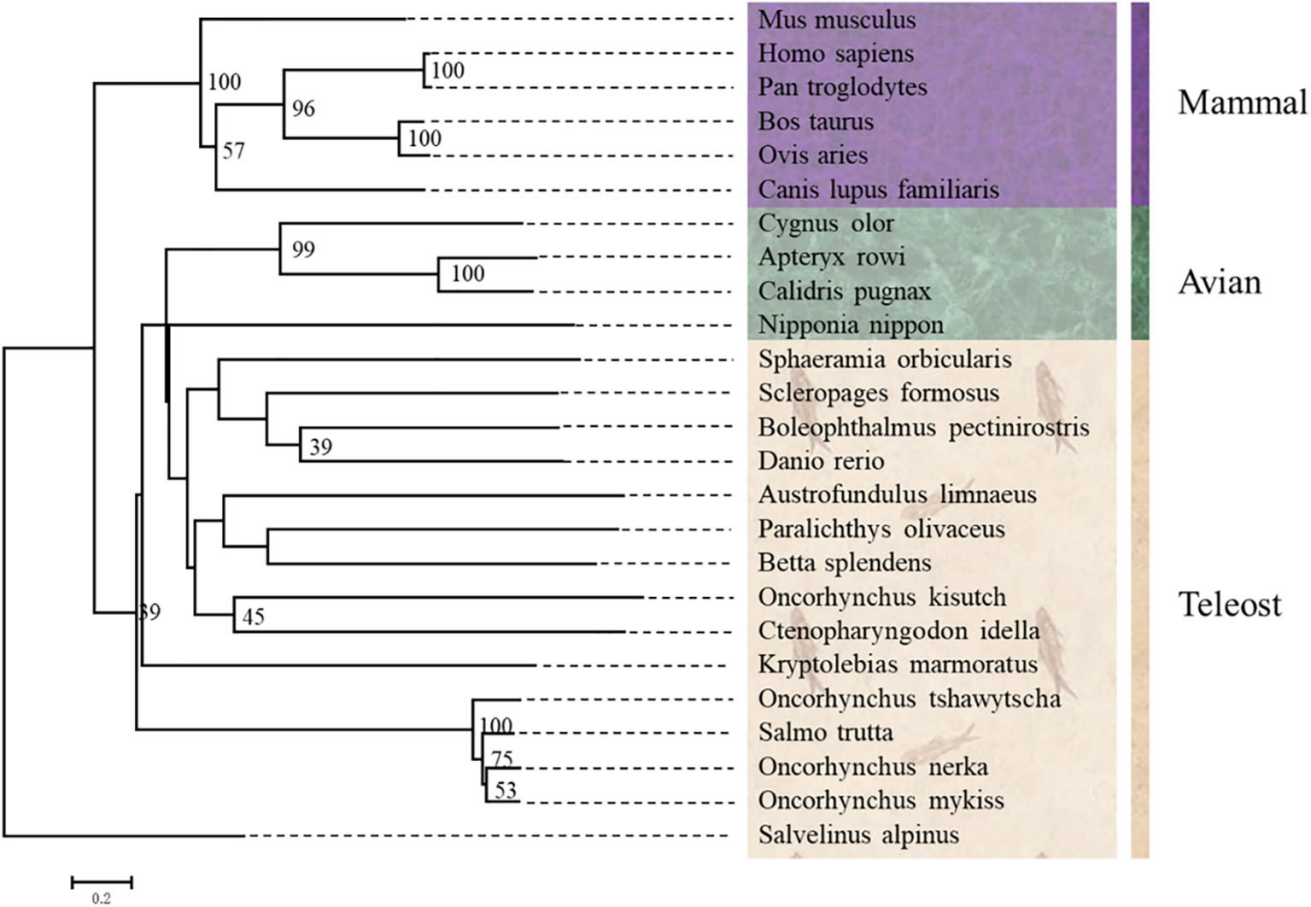
Phylogenetic trees of ICAM-1 gene from *Oncorhynchus mykiss* and other vertebrates were constructed using MEGA7.0 with the neighboring-joining (NJ) method.

Multiple alignments analysis showed that the deduced amino acids of ICAM-1 from *Oncorhynchus mykiss* had high similarity identities to other fish reported ICAM-1. The similarity of ICAM-1 went from 69 to 89% in teleost fish, but only 25–30% in mammals. To further clarify the location of ICAM-1 in phylogenetic evolution, we used NJ methods to construct a phylogenetic tree using NJ method. As expected, trout ICAM-1 represented the highest similarity with *Oncorhynchus tshawytscha* (89%), followed by *Salvelinus alpinus* (85%), but low identities to ICAM-1 of *Ctenopharyngodon idella* (49%) (**Figure 2**). However, as to mammal clade, O*ncorhynchus mykiss* ICAM-1 showed limited similarity to the mammalian ICAM-1 (**Figure 2**). Furthermore, the exon and intron organization of ICAM-1 was analyzed in four teleost fishes and human, which showed that rainbow trout existing 12 exons and 11 introns shared more similar with *Oncorhynchus tshawytscha* and *Salvelinus alpinus* (**Supplementary Figure 1**).

### ICAM-1 Differential Expression in Different Tissues of Rainbow Trout

**Figure 3:**
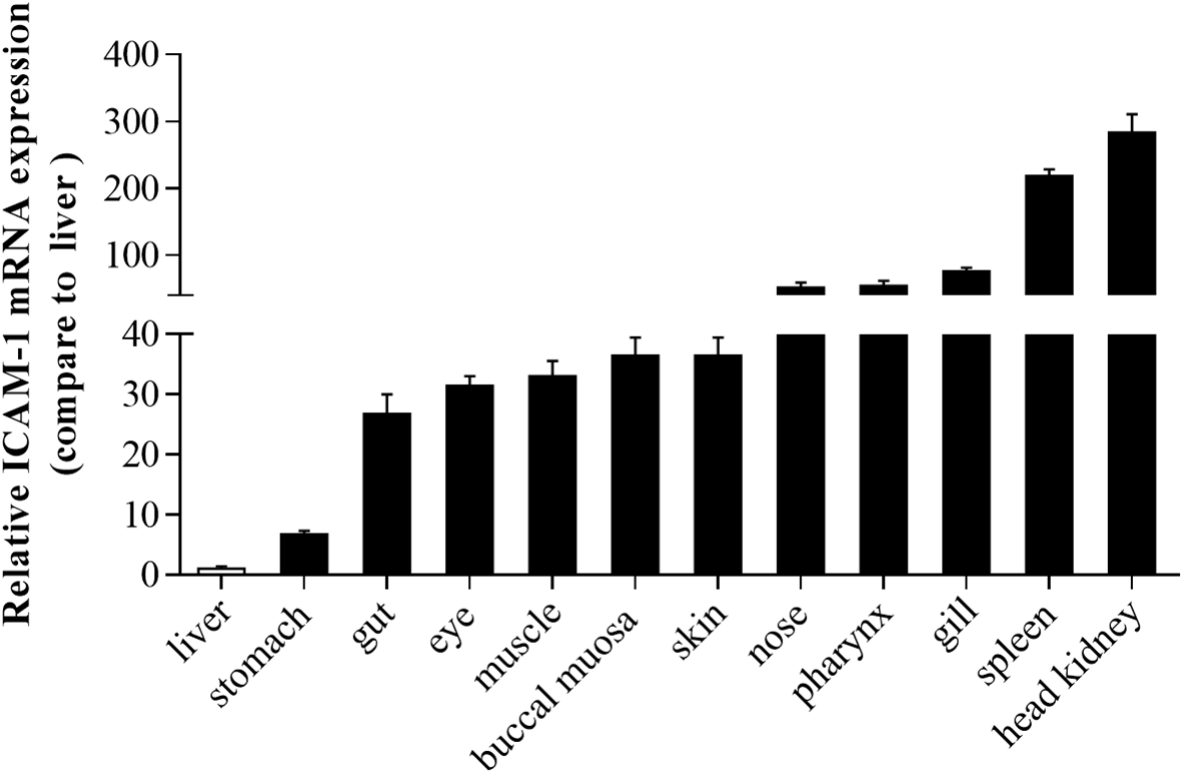
The expression pattern of ICAM-1 mRNA in different tissues of *Oncorhynchus mykiss.* Relative expression of ICAM-1 was detected in different tissues compared to the liver (n=8).

Using RT-qPCR, the expression of ICAM-1 was analyzed in different tissues including liver, stomach, gut, eye, muscle, buccal mucosa, skin, nose, pharynx, gills, spleen, and head kidney. The ICAM-1 mRNA was extensively and differently expressed in various tissues (**Figure 3**). The lowest expression level of ICAM-1was shown in the liver, followed by the stomach. Importantly, the highest expression of ICAM-1 was detected in the spleen and head kidney when compared to that of liver. Interestingly, in some other mucosal tissues including gills, pharynx, nose, skin, buccal mucosal, eye, gut, and non-mucosal tissue (muscle), the relatively high expression was also detected in comparison with that of the liver (**Figure 3**).

### Morphological Changes and ICAM-1 mRNA Expression After IHNV Infection

**Figure 4:**
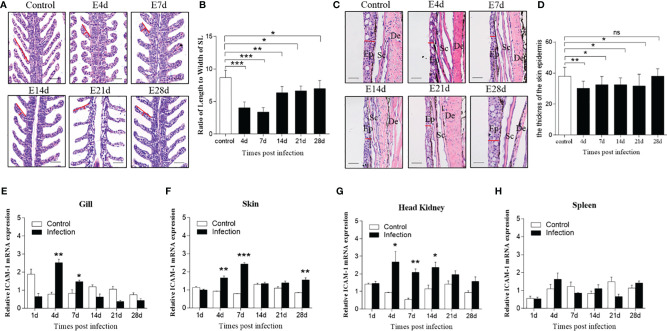
Morphological changes of skin and gills and the expression level of ICAM-1 mRNA responses to IHNV in different tissues. **(A)** Hematoxylin and eosin (HE) stains of gills from mock-infected fish, and 4, 7, 14, 21, and 28 d after IHNV challenge, respectively. Red line indicates length or width of the secondary lamellae of (SL) gills. Scale bar, 40 μm. **(B)** The length-width ratio of gill SL from control and infected fish (n=6 per group). **(C)** Histological examination by HE staining of skin from trout infected with IHNV after 4, 7, 14, 21, and 28 days and uninfected fish. Red line represents the thickness of the skin epidermis. Ep, epidermis; Sc, Scales; De, dermis. Scale bar, 40 μm. **(D)** The statistics of the skin epidermis thickness including uninfected and infected fish (n=6 per group). **(E–H)** Fold change of ICAM-1 mRNA expression in gills **(E)**, skin **(F)**, head kidney **(G)**, and spleen **(H)** at 1, 4, 7, 14, 21, and 28 d post infection compared to control group (n=6 per group). Statistical analysis was performed by unpaired Student’s t test. ns P > 0.05, *P < 0.05, **P < 0.01, ***P < 0.001. Data are representative of at least three independent experiments (mean ± SEM).

To assess the differential expression of ICAM-1 after viral challenge, we successfully developed the infected model of IHNV represented by the obvious appearance of pathological changes characterized by darkening of the skin, pale gills, exophthalmia, and petechial hemorrhage (**Supplementary Figure 2A**). Moreover, RT-PCR analyses detected the expression of the IHNV-G gene in gills, skin, spleen, and head kidney at 4 and 7 d after IHNV infection, which further indicated that fish were successfully invaded with IHNV (**Supplementary Figure 2B**). Furthermore, histological studies were implemented to show morphological changes in trout gills and skin after infected with IHNV. Here, upon IHNV infection, extremely significant changes of gills’ histology were detected at 4, 7, and 14 d post infection, and moderate changes of gill damage were observed at 21 and 28 d after infection, when compared with that of control fish, as evidenced by wider and shorter secondary lamellae with edema (**Figures 4A, B**). In addition, the thickness of skin epidermis was decreased obviously at 4, 7, 14, and 21 d after IHNV infection, whereas recovered at 28 d post infection (**Figures 4C, D**). Similarly, significant tissue damage could be observed in the head kidney and spleen after IHNV infection in comparison with the control group (**Supplementary Figure 2C**).

With regard to the expression level of ICAM-1 after challenge, by RT-qPCR, there were significant changes detected in all four tissues including the gills, skin, head kidney, and spleen at different time points post IHNV infection. Similarly, significantly upregulated expression was shown in the gills (~2.5 fold), skin (~1.8 fold and ~3 fold), and head kidney (~2.8 fold and ~3.4 fold) at days 4 and 7 post infection, but in the spleen (~1.8 fold) only at 4 d after infection compared to control fish (**Figures 4E–H**). In addition, although moderate change, the expression of ICAM-1 mRNA was also upregulated in the skin (~2 fold) at 28 d and in the head kidney at 14 d (~2 fold) after IHNV challenge, respectively (**Figures 4F, G**).

### Morphological Changes and ICAM-1 mRNA Expression After *F. columnare* Infection

**Figure 5:**
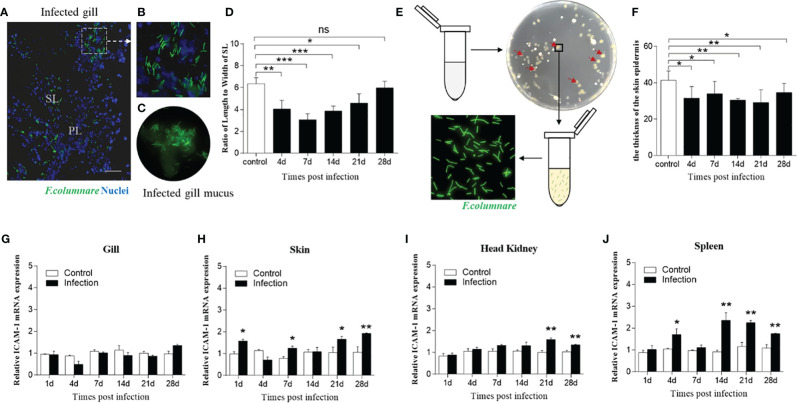
Morphological and molecular differences after challenge with *F. columnare*. **(A)** Immunofluorescence staining of gills infected by *F. columnare* labeled with GFP (green fluorescent protein). Green represents *F. columnare*; blue indicates nuclei. SL, secondary lamellae; PL, primary lamellae. **(B)** Partial enlarged view of **(A)**. **(C)** *F. columnare* were detected in the infected gill mucus. **(D)** The length-width ratio of gill SL from fish uninfected and infected with *F. columnare* (n=6 per group). **(E)** *F. columnare* in the infected skin mucus propagated in the culture plate and monoclonal colony were selected to authenticate the present of *F. columnare* (green). **(F)** The thickness of skin epidermis at 4, 7, 14, 21, and 28 d post infection (n=6 per group). **(G–J)** ICAM-1 gene was modulated by *F. columnare* infection in the gills **(G)**, skin **(H)**, head kidney **(I)**, and spleen **(J)** at days 1, 4, 7, 14, 21, 28 post infection (n = 6 per group). Data are expressed as mean fold increase in expression. Statistical analysis was performed by unpaired Student’s t test. ns P > 0.05, *P < 0.05, **P < 0.01, ***P < 0.001. Scale bar, 40 μm. Data are representative of three independent experiments (mean ± SEM).

We next assessed whether bacterial infection triggered prominent changes of ICAM-1 mRNA expression; thus, trout were immersed with *F. columnare* G_4_ strain marked with GFP. Immunofluorescence results demonstrated the successful invasion of *F. columnare* into the trout gills after bath infection when compared with that of control trout (**Figures 5A, B**). From the lapping liquid supernatant of infected gills, the *F. columnare* was clearly observed by immunofluorescence microscopy (**Figure 5C**). Thereafter, the ratio of length to width of secondary lamellae was detected from control and infection gills based on the invasion of *F. columnare*, which showed remarkable changes characterized by shorter and thicker of secondary lamellae at 4, 7, 14, and 21 d after infection (**Figure 5D** and **Supplementary Figure 3**). Otherwise, the tissue homogenates of trout skin from infected fish were cultured on Shieh agar. After incubation for 48 h, a large number of colonies were found on the Shieh agar plate, which were root-like, flat, and yellow in the center (**Figure 5E**). Then, a single colony from the plate of homogenates was selected and cultured on fluid Shieh medium at a 28°C incubator, *F. columnare* was observed under immunofluorescence microscopy (**Figure 5E**). By HE staining, morphological changes were observed in the skin on the basis of *F. columnare* invasion, which showed a significant decrease of the thickness of the skin epidermis, especially at 14 and 21 d post infection, and then recovered at 28 d post infection (**Figure 5F** and **Supplementary Figure 3**).

Next, we detected the ICAM-1 mRNA expression level in four tissues after *F. columnare* invasion, which showed moderately upregulated expression after infection in the mass. In this regard, the expression of ICAM-1gene was significantly increased in the skin, head kidney, and spleen at 21 and 28 d after bacterial infection (**Figures 5H–J**). Although no significant expression of ICAM-1 in the gills was detected after challenge at all time points, mildly increased expression of ICAM-1 gene was detected at 28 d post infection (**Figure 5G**). Taken together, these results indicated that ICAM-1 might play an important role in mucosal immune response to bacterial infection.

### Morphological Changes and ICAM-1 mRNA Expression After *I. multifiliis* Infection

**Figure 6:**
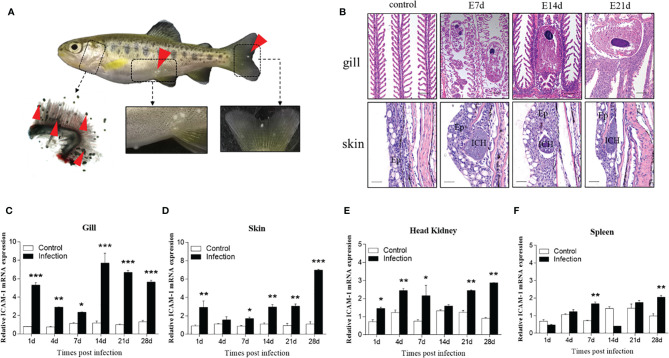
Changes induced by *I. multifiliis* infection. **(A)** The phenotype of an infected rainbow trout. Red arrowheads point to Ich trophonts. Details were shown in the gills, skin, and fin. **(B)** Hematoxylin/eosin staining of gills and skin from infected fish at different days. Ep, epidermis; ICH, *I. multifiliis*. Scale bar, 40 μm. **(C–F)** The ICAM-1 mRNA expression level of the gills **(C)**, skin **(D)**, head kidney **(E)**, and spleen **(F)** at 1, 4, 7, 14, 21, and 28 days from infected and control trout (n = 6 per group). Data are expressed as mean fold increase in expression. Statistical analysis was performed by unpaired Student’s t test. *P < 0.05, **P < 0.01, ***P < 0.001. Data are representative of three different independent experiments (mean ± SEM).

Then, to evaluate the ICAM-1 response to parasitic challenge, fish were exposed to *I. multifiliis*. After that, *I. multifiliis* were clearly observed in the gills, skin, and fins of infected fish manifested as little white dots (**Figure 6A**). Using histological studies, Ich parasites were easily detected in the secondary lamellae of gills and the skin epidermis of trout at 7, 14, and 21 d after infection (**Figure 6B**).

Similarly, we tested the ICAM-1 mRNA expression in the gills, skin, head kidney, and spleen after *I. multifiliis* infection. Our results showed that the ICAM-1 gene was upregulated after infection in the four tissues (**Figures 6C–F**). Similar tendencies to increase in expression of ICAM-1 were observed in all tested tissues. However, the most significant increased expression of ICAM-1 gene was detected in the gills and skin at 14, 21, and 28 d after challenge (**Figures 6C, D**). When compared to the gills and skin, the expression level of ICAM-1 was lower increased after infection (**Figures 6E, F**). When compared to the viral and bacterial infections, the higher expression level of ICAM-1 was detected in skin and gills after parasitic infection. Our finding demonstrated increased ICAM-1 expression levels in the tested tissues after parasitic infection.

## Discussion

ICAM-1 is a critical molecule that allows cells to adhere to each other, as well as to extracellular matrix molecules, thereby regulating leukocyte recruitment to inflammation sites during pathogen invasion. Several studies have characterized the sequence of the ICAM-1 gene in mammals (e.g., human, murine, and canine models) (8, 25, 36, 37), and the cloned sequences exhibit limited similarity to the human sequence (55–65%). However, very few studies have characterized the structure and functions of ICAM-1 in teleost fish, as well as its role in regulating the mucosal immune response against pathogens. In this regard, our study was the first to clone the sequence of ICAM-1 in rainbow trout, a teleost species, after which we analyzed the differential expression of the ICAM-1 gene in different tissues. Moreover, our study investigated the morphological changes of the gills and skin tissues and the expression levels of the ICAM-1 gene in rainbow trout upon infection with IHNV, *F. columnare*, and *I. multifiliis*.

The predicted amino acid sequence of ICAM-1 indicated that the coding sequence consisted of a signal peptide, free cysteine residues, an Ig-like domain, and a transmembrane region, which shared a high degree of similarity with other predicted ICAM-1 sequences of other teleost fish, including *Oncorhynchus tshawytscha* (89%) and *Salvelinus alpinus* (85%). However, when compared to ICAM-1 sequence of *Ctenopharyngodon idella* first characterized in fish (31), there was only 49% similarity in the sequence identity. Additionally, analysis of the exon and intron organization of ICAM-1 indicated that rainbow trout had a high similarity with *Oncorhynchus tshawytscha* and *Salvelinus alpinus* compared to that of human and grass carp (31). This may be because *Oncorhynchus mykiss* belongs to the Salmoniformes order and is more closely related to the Salmonidae family. In contrast, when compared with the mammalian ICAM-1 structure (2), there are several regions, including the signal peptide and transmembrane region, that followed conserved principles in the evolution process, whereas the number of Ig-like domains related to the ICAM-1 function was different, suggesting that the function of ICAM-1 might have gradually improved throughout vertebrate evolution. Previous studies cloned and analyzed the ICAM-1 gene in several vertebrate species (2, 24, 25, 36, 37). Here, the BLAST search algorithm was used to analyze the similarity between the predicted sequence of ICAM-1 amino acids based on cDNA sequences of rainbow trout with those of other species. Our results indicated that rainbow trout ICAM-1 sequences had a higher degree of similarity with those of *Oncorhynchus tshawytscha* (89%) and *Salvelinus alpinus* (85%) compared to other teleost fish.

It is worth noting that ICAM-1 is expressed in immune, endothelial, and epithelial cells in mammals (8), suggesting the extensive distribution of ICAM-1 in different tissues. To support this hypothesis, our study conducted RT-qPCR to detect the differential expression of ICAM-1 mRNA in different tissues of rainbow trout including skin, gills, gut, stomach, eye, buccal mucosa, nose, pharynx, liver, spleen, head kidney, and muscle. Among these, the ICAM-1 was most abundantly distributed in the spleen and head kidney, whereas lower expression levels were identified in the liver and stomach. This was consistent with previous report that not all endothelial and epithelial cell types expressed ICAM-1 *in vivo*, for example, hepatic portal veins and arteries (38) and gastric epithelial cells (39). Mucosal tissues including skin, gills, gut, eye, buccal mucosa, nose, and pharynx also exhibited relatively high expression levels of ICAM-1 in rainbow trout, implying that ICAM-1 might play an important role in immune sites. However, further research is needed to clarify the function mechanism played by ICAM-1 in mucosal immune tissues. However, the highest expression of ICAM-1 was detected in the liver of grass carp, while low expression was noted in head kidney, spleen, and gills (31), which was in contrast with that of rainbow trout in the present study. The reason might be that different diet and habitat were present in the two fishes. Rainbow trout is carnivorous cold-water fish, while grass carp is herbivorous warm-water fish. Moreover, in the ICAM-1 sequence identity, low similarity was found between grass carp and rainbow trout, which might lead to the differential expression of ICAM-1 in the two fishes.

Infectious hematopoietic necrosis virus (IHNV) is an economically important pathogen causing infectious hematopoietic necrosis and high mortalities in salmonid fishes such as Atlantic salmon and rainbow trout (40). When fish were infected with IHNV, the virus primarily proliferates into the hematopoietic tissues such as the spleen and head kidney of fish, and then some typical clinical signs and symptoms, including darkening of the skin, pale gills, exophthalmia, petechial hemorrhages, empty gut, and ascitic fluid, were induced (40). As expected, the same symptoms were observed in rainbow trout infected with IHNV in our studies. RT-PCR analyses detected the expression of the IHNV-G gene in gills, skin, spleen, and head kidney at 4 and 7 d after IHNV infection. Moreover, the morphology of the spleen, head kidney, skin, and gills in trout exhibited evident lesions after IHNV challenge. Thus, our morphological analyses demonstrated the occurrence of serious necrosis in the spleen and head kidney, which was coupled with a decreased length and increased width of gills’ secondary lamellae (SL) in the infected trout compared to the control fish. Additionally, epidermis thickness was also decreased after IHNV infection. Overall, morphological changes observed herein were interpreted as direct evidence of disease onset caused by IHNV infection in rainbow trout. Moreover, ICAM-1-deficient mice exhibited a normal development but displayed abnormal inflammatory and immune functions (41). Furthermore, Orf virus (42) and influenza virus (28) infection enhanced the expression of ICAM-1 in mammals. Based on the IHNV infection model, ICAM-1 mRNA expression was similarly upregulated in gills, skin, spleen, and head kidney, suggesting that ICAM-1 might be involved in the immune response against IHNV. Importantly, significantly higher expression levels of ICAM-1 were detected in mucosal tissues such as the skin and gills, especially at the early stages (i.e., 4 d and 7 d) of the infection. Interestingly, similar results were found at 4, 7, and 14 d in the head kidney and at 4 d in the spleen, which might be the reason that IHNV targets the head kidney and spleen for propagation. Numerous inflammatory factors might be induced at 4 and 7 d after IHNV infection, which further affect the expression of ICAM-1 and histopathologic changes in the gills and skin. In conclusion, these results indicated that ICAM-1 might play an important role in mucosal immune response to viral infection in teleost fish. Unexpectedly, we found that ICAM-1 expression level in infected fish was much lower than that in control fish on the 1st day of IHNV infection in the gills. Compared to that of other tissues, the reason might be that the gills, as the respiratory organ, were easily affected by aquatic environmental factor.

Similarly, we constructed a bacterial infection model of rainbow trout using *F. columnare* G_4_ marked with a green fluorescent protein. Our immunofluorescence analyses identified *F. columnare* in the gills’ mucus sections of infected trout. Moreover, the tissue homogenates of trout skin from infected fish were cultured on Shieh agar, where *F. columnare* colonies were detected. The GFP-marked *F. columnare* cultured in fluid Shieh medium from the colony was then detected using immunofluorescence microscopy. Overall, all data indicated that trout were successfully infected with the *F. columnare* G_4_ strain. Afterward, we analyzed the morphological changes of gills and skin upon *F. columnare* infection, which showed similar results to those of IHNV-infected fish. In mammals, previous studies have shown that LPS cannot induce ICAM-1 expression in the intestinal epithelial cells (8), but enhanced ICAM-1 expression in the abnormal cells, such as senescent endothelial cells and cancer cells (43, 44). Importantly, ICAM-1 plays a vital role in airway inflammation and bacterial clearance (26). In the present study, we found that the expression level of ICAM-1 was upregulated after *F. columnare* challenge, especially in the skin, but moderate increased expression in the head kidney and spleen. Interestingly, we found that specifically downregulated expression was detected on day 7 of infection in the spleen. One reason similar with *I. multifiliis* infection is that innate immune response was gradually decreased and adaptive immune response was induced from 7 d after bacterial infection. Additionally, it has previously been reported that the absence of ICAM-1 seems to protect mice against lethal septic shock (45). Thus, the downregulated expression of ICAM-1 at 7 d might play a role in protecting fish from septic shock, but this hypothesis warrants to be determined by further research. In conclusion, an induction of ICAM-1 expression by bacterial infection might indicate an important mucosal immune role of this receptor during bacterial infection, but the mechanism played by ICAM-1 remains to be understood.

Rainbow trout were also challenged with *I. multifiliis* to detect the differential expression of the ICAM-1 gene in response to parasitic infection. Clinical manifestations (e.g., white spots) appeared on the surface of the fins, gills, and skin, and histological analyses suggested a successful parasitic infection in rainbow trout. As to the expression of ICAM-1, *Toxoplasma gondii* results in increased ICAM-1 expression in the jejunum of rats after infection (46), and ICAM-1 expression increased in the skin of mice after vaccination with *Schistosoma mansoni* (47). Similarly, we also found an enhanced expression of ICAM-1 in the gills, skin, head kidney, and spleen of trout infected with *I. multifiliis*. It is worth noting that the higher expression level of ICAM-1 was observed in the gills and skin after parasitic infection compared to that of viral and bacterial infections. The reason might be that *I. multifiliis* firstly invaded and damaged the mucosal tissues such as gills (48, 49), skin (50), buccal mucosa (34), and nose (35) after infection. Interestingly, we found that the expression of ICAM-1 was periodic after Ich infection. The main reason might be that *I. multifiliis*, as a mucosal pathogen, could easily invade mucosal tissues of fish, such as gills and skin, and ICAM-1 acts as a receptor for pathogens; thus, a higher expression of ICAM-1 and innate immune responses could be induced at a short time. Over time, ICAM-1 was upregulated at 14 d to induce adaptive immune response by recruiting immune cells. Then, with the enhancement of adaptive immunity, *I. multifiliis* was gradually expelled from the body, which resulted in the expression of ICAM-1 gradually decreasing from 14 d to 28 d. Unexpectedly, we found expression of ICAM-1 decreased significantly in spleen and head kidney at day 14, which could be explained by the immune response of spleen being hysteretic compared to that of gills. Yet, similar to virus and bacteria, ICAM-1 is also critical for mucosal immune response against parasitic infection in teleost fish.

In conclusion, our study was the first to characterize the full-length cDNAs of ICAM-1 from *Oncorhynchus mykiss*, which shared similar characteristics with their mammalian counterparts. Tissue differential expression analysis indicated that this gene was most highly expressed in the spleen and head kidney, followed by a few mucosal tissues such as the gills and skin. In contrast, the liver and stomach exhibited lowest ICAM-1 expression level. Furthermore, to investigate the role of ICAM-1 in the immune response of rainbow trout, three infection models including virus (IHNV), bacteria (*F. columnare*), and parasite (*I. multifiliis*) were successfully constructed, represented by pathological changes of tissues and typical clinical manifestations in the present study for the first time. Further, our pathogen challenge experiments revealed that the expression of the ICAM-1 gene was significantly upregulated not only in the head kidney and spleen but also in the gills and skin after IHNV challenge. In contrast, almost all remaining tissues examined herein exhibited an enhanced ICAM-1 expression in response to bacterial and parasitic infections. Of note, our results showed that ICAM-1 expression was lower after bacterial infection when compared to that of viral and parasitic infection, indicating bacteria might not trigger as strong immune response as those of virus and parasite induced by ICAM-1, which could be explained that bacterial LPS might not induce the strong expression of ICAM-1. Combined, our results in this study demonstrate that ICAM-1 plays an important role in the mucosal immune response to viral, bacterial, and parasitic infections in teleost fish.

## Data Availability Statement

The raw data supporting the conclusions of this article will be made available by the authors, without undue reservation.

## Ethics Statement

Animal procedures were approved by the Animal Experiment Committee of Institute of Hydrobiology, Chinese Academy of Sciences.

## Author Contributions

XZ and W-GK performed most of the experiments and wrote the manuscript. XZ analyzed the data. G-FC, FD, J-FC, G-KH, Y-LS, and C-JQ helped with most of the experiments. ZX designed the experiments and revised the manuscript. All authors contributed to the article and approved the submitted version.

## Funding

This work was supported by grants from the National Natural Science Foundation of China (U1905204, 32073001, and 31873045) and grant from Key Laboratory of Sichuan Province for Fishes Conservation and Utilization in the Upper Reaches of the Yangtze River, Neijiang Normal University (NJTCSC01).

## Conflict of Interest

The authors declare that the research was conducted in the absence of any commercial or financial relationships that could be construed as a potential conflict of interest.

## Publisher’s Note

All claims expressed in this article are solely those of the authors and do not necessarily represent those of their affiliated organizations, or those of the publisher, the editors and the reviewers. Any product that may be evaluated in this article, or claim that may be made by its manufacturer, is not guaranteed or endorsed by the publisher.
