# Supplementary material for: Molecular Characterization and Expression Analysis of Intercellular Adhesion Molecule-1 (ICAM-1) Genes in Rainbow Trout (Oncorhynchus mykiss) in Response to Viral, Bacterial and Parasitic Challenge
Source: Front Immunol. 2021 Aug 20;12:704224. doi: 10.3389/fimmu.2021.704224 (PMC8417878; doi:10.3389/fimmu.2021.704224)
Supplement: Supplementary file 1 [file DataSheet_1.docx]

**Molecular characterization and expression analysis of intercellular adhesion molecule-1 (ICAM-1) genes in rainbow trout (*Oncorhynchus mykiss*) in response to viral, bacterial and parasitic challenge**

Xue Zhai^1¶^, Wei-Guang Kong^1¶^, Gao-Feng Cheng^1^, Jia-Feng Cao^1^, Fen Dong^1^, Guang-Kun Han^1^, Yan-Ling Song^1^, Chuan-Jie Qin^3^ and Zhen Xu^2*^

^1^Department of Aquatic Animal Medicine, College of Fisheries, Huazhong Agricultural University, Wuhan, Hubei 430070, China

^2^ State Key Laboratory of Freshwater Ecology and Biotechnology, Center for Fish Biology and Fishery Biotechnology, Institute of Hydrobiology, Chinese Academy of Sciences, Wuhan, Hubei, 430072, China ^¶^ These authors contributed equally to this work.

^3^ department of life science, Key Laboratory of Sichuan Province for Conservation and Utilization of Fishes Resources in the Upper Reaches of the Yangtze River, Neijiang Normal University, Neijiang, China

^*^Corresponding Author: zhenxu@ihb.ac.cn.

**Supplementary materials**

**Supplementary Table 1**. Primers used in this study.

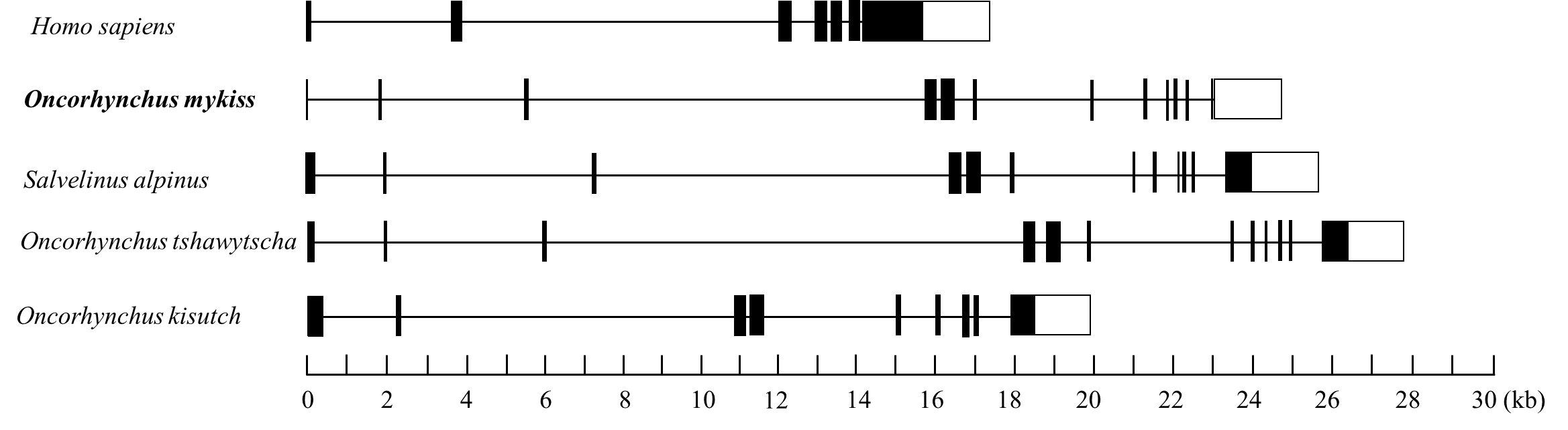


**FIGURE S1 | Exon and intron organization of ICAM-1 of *Homo sapiens, Oncorhynchus mykiss, Salvelinus alpinus, Oncorhynchus tshawytscha, Oncorhynchus kisutch*. White boxes, black boxes, and solid lines represent the untranslated regions, exon and intron, respectively.**


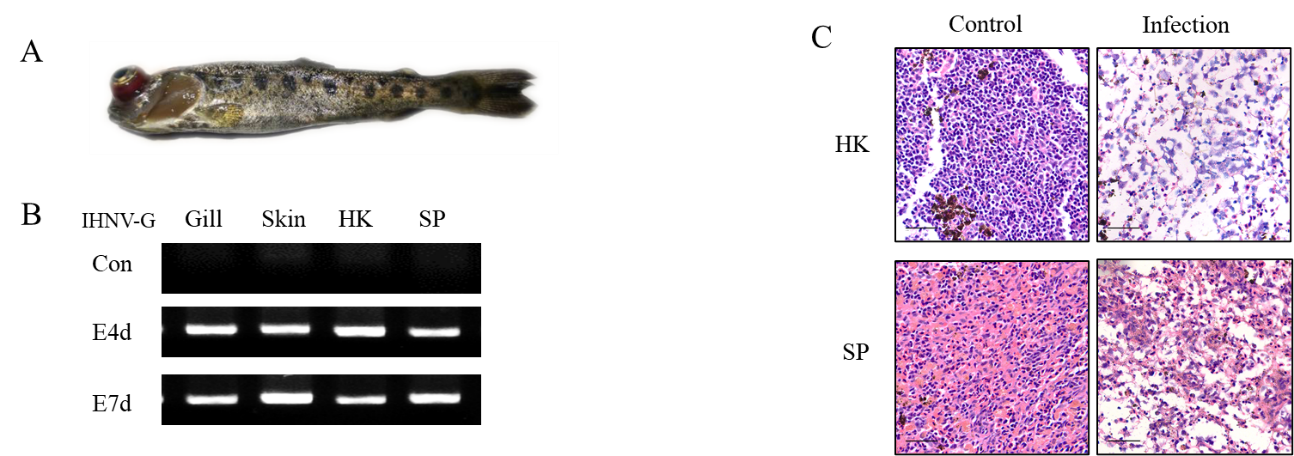


**FIGURE S2 |** Clinical manifestations, detection of IHNV-G gene and morphological changes of head kidney and spleen after IHNV infection. **(A)** Typical gross appearance such as darkening of the skin, pale gills, exophthalmia, petechial haemorrhages. **(B)** PCR analyses detected the expression of the IHNV-G gene in gills, skin, spleen, and head kidney at 4 and 7 d after IHNV infection. **(C)** Morphological changes of head kidney and spleen in the control and infection fish. HK, head kidney; SP, spleen. scale bars, 40 µm.


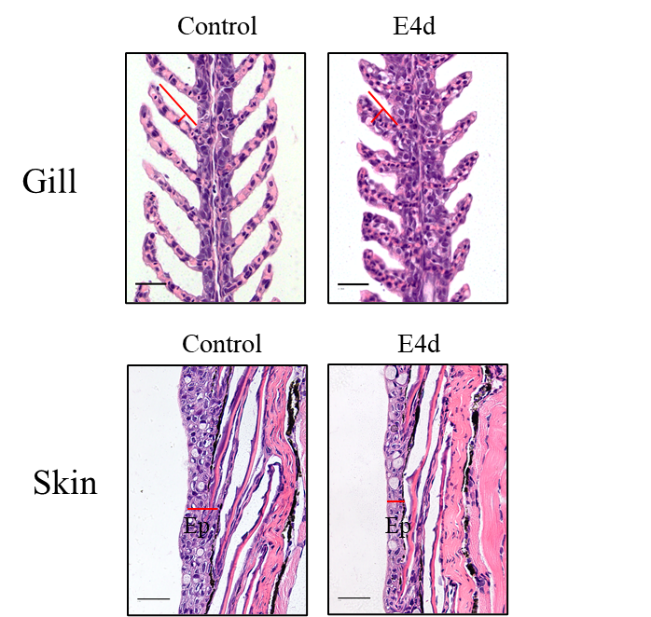


**FIGURE S3 |** Morphological changes of skin and gills in the control fish and 4d-infected fish after *F. columnare* infection.
